# Safety of topical thrombins: the ongoing debate

**DOI:** 10.1186/1754-9493-3-21

**Published:** 2009-09-04

**Authors:** Christopher Lomax

**Affiliations:** 1 Adjunct Assistant Professor of Pharmacy, University of Southern California, School of Pharmacy, Los Angeles, CA 90033, USA

## Abstract

Until recently, only bovine-derived thrombin was available for use as a stand-alone topical hemostat or as a component of other hemostatic devices. Concerns over a number of case reports of immune-mediated coagulopathies associated with the use of bovine-derived thrombin resulted in a United States Food and Drug Administration warning letter being issued in 1996 and the later addition of a boxed warning ("Black Box Warning") to all bovine-derived thrombin products. Since 2007, both a human-pooled plasma thrombin product and a recombinant thrombin have entered the market.

With the addition of these two products to the topical thrombin class, a unique situation has developed in which only a single member (bovine-derived thrombin) within the class carries the Food and Drug Administration's strongest cautionary language about possible adverse events related to an agent's use. Neither the human-pooled plasma thrombin nor the recombinant thrombin products have a boxed warning; although, the human-pooled plasma product does include a precaution/warning about infectious agent transmission - a warning common to products derived from human sources. This report will address this unique situation and the impact, clinical and non-clinical, that thrombin choice may have. Since alternatives are now available, institutions may need to revisit their formulary choice of thrombin preparation, taking into consideration the potential risks associated with bovine-derived products.

## Findings

Annually, between 370,000 and 500,000 patients in the United States are exposed to topical bovine thrombin (bThrombin) for surgical hemostasis [1]. Thrombin is a clotting factor, the main role of which is to convert soluble fibrinogen into insoluble strands of fibrin, the foundation of a blood clot [2]. Since thrombin's first clinical applications in the 1940s, its ease of use and utility in general, cardiovascular, orthopedic, gynecologic, and neurologic surgical procedures, either as standalone agent or in combination with surgical sponges, has become a mainstay in surgical hemostasis. Beginning in the 1950s, however, cases of immune reactions due to bThrombin exposure have continued to appear in the literature. In 1990, the first description of bThrombin antibodies with cross-reactivity to native coagulation factors was reported, ultimately resulting in an FDA warning letter regarding occasional hemostatic abnormalities and finally, the inclusion of a boxed warning in the package insert of bThrombin [2].

To date, over 100 cases of bThrombin-associated acquired factor V and thrombin (factor IIa) antibodies have been reported in the literature and have been associated with various adverse events. [2] A search of PubMed and Medline databases for English-language reviews and case reports has demonstrated that new cases of immune-mediated coagulopathies (IMC) have continued to emerge since 2001 [1,3-6]. Given the inconsistent reporting of involved agents, confounding diagnoses, and a low index of suspicion in the medical field, the number of affected patients may be under-reported to a significant degree [7]. These antibodies may be associated with potentially serious, and in rare instances, fatal hemostatic abnormalities, and clinicians need to be aware that these coagulopathies may result from the use of bThrombin. The manifestations of bThrombin-associated IMC can range from abnormal coagulation studies without clinical sequelae to hemorrhagic immune-mediated coagulopathies requiring reoperation. The intent of this short report is to increase awareness of the unique nature of the topical thrombin class as it pertains to the immunogenic profiles of the agents in the class, and to briefly describe clinical and non-clinical aspects of thrombin choices.

Thrombin from bovine sources is the oldest of its class (Thrombin-JMI, GenTrac, Inc, Middleton, WI) and is derived through a conversion reaction in which bovine prothrombin is activated by tissue thromboplastin in the presence of calcium chloride. The resulting product is then chromatographically purified and undergoes ultrafiltration to reduce factor V and its fragments [8]. Two new products have recently been approved and were developed to address concerns centering on the immunogenicity of the bThrombin product. The first to become available in 2007 was a human-plasma-derived thrombin product (pdThrombin) (Evithrom, OMRIX biopharmaceuticals, Kiryat Ono, Israel); followed in early 2008 by the first recombinant human thrombin product (rhThrombin) to be approved by the United States Food and Drug Administration (FDA) (Recothrom, ZymoGenetics, Inc., Seattle, WA).

PdThrombin is derived from pooled plasma obtained from FDA-licensed plasmapheresis centers and is antigenically distinct from bovine thrombin in both amino acid sequence and glycosylation patterns [2]. RhThrombin is produced by Chinese hamster ovary (CHO) cells from precursor recombinant prethrombin-1 derived from cell culture and has an identical amino acid sequence and disulfide-bonding pattern to human thrombin derived from plasma. In separate phase 3, comparative clinical trials, rhThrombin versus bThrombin and pdthrombin versus bThrombin found the newest agents equal in efficacy to bThrombin with a similar adverse event profiles [9,10]. Both products did, however, demonstrate a statistically significant lower incidence of anti-product antibody formation compared to bovine thrombin. In the only study of its type to date, rhThrombin was found to be minimally immunogenic in patients with preexisting antibodies to bThrombin products and the authors concluded that rhThrombin could be used safely in patients with or without preexisting antibovine product antibodies [11].

Recent studies have demonstrated that the current ultrafiltration methods for bThrombin appear to remove significant amounts of bovine factor V as well as other antigens and viruses [12]. These manufacturing changes were designed to remove immunogenic contaminants that are believed to be at least as immunogenic as the bThrombin itself [8,12]. However, this still represents only a reduction in factor V contamination, not complete removal, and the clinical significance of this ongoing contamination is unknown. (The remote possibility still exists that anti-factor V antibodies may be generated following the use of bThrombin and the black box warning remains [13]).

A recent paper by Singla et al in the Journal of the American College of Surgeons demonstrated that 15.6% of patients with documented or likely exposure to bThrombin were positive for anti-bovine thrombin antibodies up to 3 years post-exposure [11]. This finding is significant because patients treated with older formulations may remain at risk for developing IMC if re-exposed to bThrombin for several years after their initial exposure. It bears repeating that the warning on bThrombin is explicit about not re-exposing patients to b-thrombin if they have antibodies to bThrombin. Unfortunately, there is no commercially available test for detecting these antibodies.

Additionally, should an IMC develop, an average hospital stay may increase in duration by a factor of 2 to 2.5 if a transfusion is required [14]. Institutions and clinicians should be aware of the potential for use of large quantities of already scarce resources such as blood and blood products, not to mention the time, discomfort, and cost of serial laboratory testing, as well as the risks of transfusion-related complications. Other costs that may or may not be reported include those resulting from imaging studies that may be repeated serially to determine the need for surgical intervention to achieve hemostasis, and reoperation. Repeat operations carry their own inherent risks and consequences to patients and institutions, since many times the costs are unrecoverable. Furthermore, in the current environment, reimbursements for adverse events are steadily being reduced, or in some cases eliminated entirely, for events and outcomes that are judged to be avoidable or preventable [15].

The unique circumstance of only a single member of the thrombin class bearing a warning of potentially serious adverse events resulting from its use may have more far-reaching implications. The *Code of Federal Regulations *Title 42 Section 482.25 (42CFR § 482.25) states that "...*The medical staff is responsible for developing policies and procedures that minimize drug errors. This function may be delegated to the hospital's organized pharmaceutical service...*" As a result, regulators in some states have created an additional expectation that drugs with black box warnings may require additional processes and systems prior to dispensing and utilization in order to minimize risks discussed in the warning. This is not to imply that black box warnings or lack of direct data prohibit the use of sound clinical judgment in prescribing. Experience, careful consideration, and sound reasoning form the basis for decisions. However, careful consideration must include the recognition of all aspects of an agent's profile, including the black box warnings advising caution and care.

## Conclusion

While the safety profile of bThrombin has generally been good, there are numerous case studies in the literature describing clinically significant defects in hemostasis, secondary to the formation of antibodies to coagulation proteins. Prior to 2007, there were no commercial alternatives to bovine-derived thrombin, but two new thrombin products have come on the market, pdThrombin and rhThrombin, neither of which carries the black box warning and, in the case of rhThrombin, appear to be safe to use in patients previously exposed to bThrombin. In this class, formulary decisions should be driven by considerations such as the potential costs of managing an acquired, bovine-associated, immune-mediated coagulopathy should one develop, including the cost of blood, immunosuppressants, and other therapeutics along with the costs of an extended hospital stay.

## Competing interests

Christopher Lomax, PharmD, has received consulting fees from ZymoGenetics.
